# Observations on Obstetric Auscultation, with an Analysis of the Evidences of Pregnancy, and an Inquiry into the Proofs of the Life and Death of the Fœtus in Utero

**Published:** 1834-01-01

**Authors:** 


*Observations on Obstetric Auscultation, with an Analysis of the Evidences of Pregnancy, and an Inquiry into the Proofs of the Life and Death of the Fœtus in Utero.* By Evory Kennedy, M.D., Licentiate of the King and Queen's College of Physicians in Ireland, Lecturer on Midwifery, &c. *With an Appendix, containing Legal Notes*, by John Smith, Esq., Barrister at Law.—Dublin, 1833. 12mo. pp. 288.

We have perused this very interesting volume with great satisfaction, and shall introduce it to the notice of our readers in the author's own language.

"If we for a moment consider the responsible task the medical adviser is occasionally called upon to perform, when desired to pronounce on the existence or absence of pregnancy; that peace of mind, domestic happiness, character, property, nay even life itself, may be sacrificed by inaccuracy in his diagnosis; we shall find ample reason to question, whether there be, in the broad field of medical science, any subject which more calls for the attention of the practitioner, as well from its importance as from the difficulty so frequently attending its investigation.

"Every medical man, at all conversant with midwifery practice, knows how often he is required to give an opinion in cases of doubtful pregnancy, by his ordinary patients, who are naturally anxious to be acquainted with their real situation; and he also well knows what dissatisfaction and want of confidence his refusal to give a decided answer on this subject frequently engenders. How much is his embarrassment increased, when he is called on to decide in those cases where private character or public justice is at stake, and in which, from their very nature, must be expected nought but concealment and misrepresentation!

"It is a matter very easily proved, that difficulties in this respect do, and by no means very unfrequently, meet us in our practice. It shall be our endeavour to explain upon what these depend, to canvass the means at present relied upon for meeting them, and to offer some in addition, which may guide and assist us in overcoming them." (P. 1.)

The truth of the above must be known to all who practise midwifery; and any effort to clear away those difficulties should be regarded with attention, and, if successful, acknowledged with gratitude. Dr. Kennedy's work is replete with valuable information, and its value is greatly enhanced by the plain and unassuming, yet forcible language, in which it is conveyed. It is impossible to read any portion of the book without feeling convinced that the doctor is describing what he has himself seen and *heard;* that he has, in fact, written from his own experience, and not from that of others.

Our author prefers dividing his subject after the French plan, and considers pregnancy under the three following heads: Simple, Compound, and Complicated. It is termed Simple Pregnancy when there exists but one fœtus; Compound, when there is more than one; and Complicated, when disease is complicated with the pregnant condition. The evidences of pregnancy are divided into two classes: first, into those ascertained through the representation of the female supposed pregnant; and, secondly, into those sensible to the medical attendant.

"Our second division will embrace a class of signs, which, from their being cognizable to the scrutiny of the examiner, and not depending upon hearsay testimony, must approach nearer to what we may term demonstrative proofs. This we shall see more distinctly when we come to examine them in detail, when we shall find them not depending, as was generally observed in the former case, upon sympathies more or less remote, but most frequently being essentially and physically caused or produced by that which we wish to detect, namely, the fœtus itself.

"To facilitate as much as possible the investigation of these symptoms, which are actually cognizable by the medical attendant, they may be divided into three classes; the tangible, or those which come within the range of a manual examination; the visible, or those which are exposed immediately to our view; and the audible, or those more recently discovered, and to detect which we are obliged to have recourse to auscultation." (P. 28.)

Passing over the common diagnostic marks of pregnancy, we shall consider those only which are derived from auscultation, begging our readers' attentive consideration of the facts related by Dr. K.

Before a stethoscopic examination be had recourse to, the female should be placed in bed, and the abdomen covered with a sheet only, as we have been frequently baffled in our attempts to discover the motion of the fœtal heart whilst the patient has been dressed, and in the erect position. Dr. K. prefers mediate to immediate auscultation, and states that,

"As the first indication of pregnancy afforded by auscultation is the *souffle,* we shall commence with the consideration of that phenomenon.

"If we examine, either with the naked ear or the stethoscope, the abdomen of the pregnant woman, we shall (provided the pregnancy be sufficiently advanced) observe a peculiar blowing or hissing sound. This sound is to be met with in almost every case, and is observed at different parts of the uterine tumour. It does not always exhibit exactly the same characters; yet these are sufficiently striking to render it cognizable in almost every case. It assumes the different varieties which Laennec describes under the term *bellows' sound*, namely, the bellows' sound, properly so called, likened by that author to the continuous murmur, similar to that of the sea, familiarly exemplified by the application of a large shell to the ear; the rasping or sawing sound, which is occasionally found so exactly imitated as to lead the listener to imagine an artisan at work quite close to him; and the musical or hissing sound, so well described by the same author. A sound, resembling the cooing of a dove, is sometimes observable, but this is comparatively rare. A more frequent peculiarity to be noticed, is, a strange drone resembling that of a bagpipe accompanying the sound, but yet without interfering with it. The most constant form we meet with, however, is a combination of the bellows or sawing with the hissing sound, commencing with one of the former, and terminating with the latter; and this is in general so protracted, that the last souffle is audible when the subsequent one commences." (P. 64.)

There are two sounds to be distinguished in examining the abdomen of pregnant women: one is termed the *souffle,* which is produced by the rush of the maternal blood into the uterus; and, as there is a greater determination to the part at which the placenta is attached, it will generally be heard in that situation, although it may sometimes be at the lateral part of the womb, "where the distribution of vessels so resembles that in the placental part of this organ."

"It is always heard either in that part of the uterus to which the placenta is or has been attached, or, as already mentioned, in the lateral parts of the uterus, where the distribution of vessels so resembles that in the placental part of this organ. These facts we have repeatedly proved by manual examination, when it has become necessary to introduce the hand into the uterus to remove the placenta, as well as by ocular demonstration after death.

"The resemblance which exists between the vascular structure in the uterus, above described, and that observed in aneurism by anastomosis, where a similar phenomenon occurs, as well as the circumstance of this sound's existing in the cow, an animal whose utero-placental structure, as we have already seen, approaches near to that of man, go a considerable length in proof of its dependence on the above cause." (P. 70.)

It is easily distinguished from the beating of the fœtal heart, by its peculiar *bellows' sound,* and by its being synchronous with the pulsation of the arterial system of the mother.

"It is necessary to mention to those unacquainted with stethoscopic phenomena, that, in their examination for the souffle, they might, without sufficient attention, be led astray by other sounds, either from the resemblance they bear to this, or the effect they have in concealing it. The respiratory murmur is sometimes conducted from the lungs across the thoracic to the abdominal parietes, and may embarrass, but can scarcely deceive us, if we be acquainted with, or prepared to expect it. The sonorous rale resembles somewhat the placental sound, and is occasionally conducted over the abdomen in the same way as the respiratory murmur. We can invariably distinguish this by its corresponding in frequency with the respiration as calculated by the heaving of the chest, whilst the souffle is synchronous with the pulse at the wrist, or, in other words, we ought, generally speaking, to count three placental sounds for one respiratory or sonorous rale. By attending to this rule, unless the respiration be very rapid indeed, it can scarcely lead us astray: but, independent of the difference of the sound which those conversant with it can at once detect, if any doubt exist, tracing it along the abdomen will in these cases set the point at rest, as, when it depends upon respiration, it becomes more and more distinct as we approach the chest, and on arriving there, its respiratory character is quite apparent." (P. 76.)

The placental *souffle* cannot be detected until after the second month, but is easily recognised at the tenth, eleventh, or twelfth week. Dr. K. relates several cases in which, although great difficulty existed in forming a diagnosis by the ordinary means, the existence of pregnancy was clearly ascertained by the stethoscope. His remarks on this branch of the subject are followed by the following very judicious advice.

"Having so far dwelt upon the placental souffle as a test of pregnancy, it must be evident that it is not intended to attach more weight to it, as such, than experience should warrant; as, although fully convinced of the benefits to be derived from it, in a diagnostic point of view, yet we are well aware, that, like all other diagnostic means, even those most relied upon, there are some difficulties to be encountered in its universal application. We find by every day's experience, that reputed discoveries demanding the attention of the public carry conviction with them, and gain permanent character, exactly in an inverse ratio to the degree of confidence and unqualified terms of recommendation, whether mistaken or exaggerated, with which they have been set forth. The object, therefore, of every man who wishes not only the advancement of science generally, but also the adoption of the views or improvements (if they really be such) which he more particularly recommends, should be to keep strictly within the bounds of moderation, stating nothing he does not know on the subject, and everything he does; and, while he clearly distinguishes in his statements between facts and opinions, to abstain from obtruding on the public views insufficiently matured, and unsupported by proof and experience." (P. 86.)

The pulsations of the fœtus can usually be distinguished at the lower part of the mother's abdomen, by applying the stethoscope midway between the anterior superior spinous process of the ilium and the umbilicus; and, as the beats are frequently more than double in number, when compared with those at the wrist of the mother, no difficulty can possibly be felt in discriminating between the two. Where these distinct evidences of fœtal circulation are to be heard, then of course all doubts regarding the existence of pregnancy are at an end; but, as there are occasional difficulties in the way, we would strongly recommend our readers carefully to peruse the whole work for a satisfactory method of overcoming them; and we would also advise those especially who are commencing the study of auscultation, and whose ears are on that account not so capable of discriminating the different sounds, to be careful that their patients are not encumbered with too much clothing; we ourselves having been repeatedly foiled when the female has had her usual dress about her.

The sound of the fœtal heart cannot, in general, be detected before the expiration of the fourth month; and even then "it is sometimes so delicate and feeble, as to render it necessary for the individual exploring to have an ear well trained to stethoscopic sounds."

The reader will find some excellent remarks on certain disordered conditions liable to be mistaken for pregnancy, where auscultation has been satisfactorily employed for the solution of the difficulty.

Dr. Kennedy, with great justice, censures the absurdity of attempting to settle questions of doubtful pregnancy by the verdict of a jury of matrons; and he gives the following remarkable instance, in which a female, convicted of a capital offence, was declared by a dozen foolish women not to be quick with child, while the contrary opinion, maintained by three surgeons, saved the child, gave the prisoner a temporary respite, and was verified by the event.

"The case was one in which a woman, named Mary Wright, was indicted before Baron Bolland, at the Norwich Assizes, March 3d, 1833, for poisoning her husband. She was clearly found guilty of the crime, although an unavailing effort to prove her insane at the time of committing it, was attempted. The prisoner's counsel put in a plea of pregnancy in bar of execution, which the judge directed the sheriff to summon a jury of matrons to investigate. Twelve married women were found; and, after being sworn, were directed to try whether the prisoner was pregnant with a quick child. After an hour's investigation, they returned a verdict that she was not quick with child. The woman was, of course, ordered for execution on the Monday following. Under these circumstances, Surgeons Scott, Crosse, and Johnson, fully alive to the absurd, although legal method, adopted to ascertain an extremely difficult point of diagnosis, and one involving the life of an individual in its accuracy, voluntarily waited upon the convict in the jail on the morning following, and having, to use the words of my friend Mr. Scott, the eminent partner of the late celebrated Dr. Rigby, 'completely stultified the verdict of the twelve discreet matrons,' and satisfied themselves that she was not only pregnant, but quick with child, they forwarded immediately to the judge of assize the following document, duly attested, and with their respective signatures appended.

"*Norwich County Jail;*
*Saturday, quarter before nine,* A.M., *March* 23, 1833.

"To the Hon. Sir William Bolland, Knt., Baron of the Exchequer, the following representation respectfully sheweth:

"'That we, the undersigned, are surgeons and accoucheurs of considerable experience in the practice of midwifery, and have repeatedly examined females in different stages of pregnancy.

"'That we have this morning strictly examined Mary Wright, a prisoner, sentenced to be executed on Monday next, for the murder of her husband; and found her between five and six months gone in pregnancy.

"'That in an apparently vigorous and healthy woman like the prisoner, and where the size of the body has regularly increased during pregnancy, we should feel ourselves bound to believe the fœtus living, unless we found some signs of its being dead.

"'That in the prisoner, Mary Wright, we find no signs of a dead fœtus; but, on the contrary, have positive evidence of its being at this time living.

"'That we do verily believe the said prisoner is above five months advanced in pregnancy, and carries in utero a living fœtus.

"'That in a case of such a nature, we desire, without delay, to submit our statement to your lordship; and if the verdict of the jury of matrons, yesterday given, that the prisoner, Mary Wright, was not quick with child, deprive the said prisoner of a reprieve until delivery *ex necessitate legis*, we humbly entreat your lordship to respite the execution of the said Mary Wright until she be delivered.'

"Of course Baron Bolland paid the attention to this document it merited, and the woman was reprieved until after her delivery." (P. 298.)

